# Anti-Inflammatory Activities of the Ethanol Extract of *Prasiola japonica*, an Edible Freshwater Green Algae, and Its Various Solvent Fractions in LPS-Induced Macrophages and Carrageenan-Induced Paw Edema via the AP-1 Pathway

**DOI:** 10.3390/molecules27010194

**Published:** 2021-12-29

**Authors:** Laily Rahmawati, Sang Hee Park, Dong Seon Kim, Hwa Pyoung Lee, Nur Aziz, Chae Young Lee, Seung A Kim, Seok Gu Jang, Dong Sam Kim, Jae Youl Cho

**Affiliations:** 1 Department of Integrative Biotechnology, Sungkyunkwan University, Suwon 16419, Korea; lyrahma0106@g.skku.edu (L.R.); wetdry20@hanmail.com (D.S.K.); leehwapyoung57@gmail.com (H.P.L.); nuraziz@skku.edu (N.A.); chaeyoung2@g.skku.edu (C.Y.L.); seung-a26@naver.com (S.A.K.); 2 Department of Biocosmetics, Sungkyunkwan University, Suwon 16419, Korea; 84701@naver.com; 3 Research and Business Foundation, Sungkyunkwan University, Suwon 16419, Korea; jangsg69@korea.kr

**Keywords:** *Prasiola japonica*, anti-inflammatory, paw edema, AP-1 pathway

## Abstract

*Prasiola japonica* possesses several biological activities. However, reports on the anti-inflammatory activities and molecular mechanisms of its different solvent fractions remain limited. In this study, we investigated the potential anti-inflammatory activities of *P. japonica* ethanol extract (Pj-EE) and four solvent fractions of Pj-EE made with hexane (Pj-EE-HF), chloroform (Pj-EE-CF), butanol (Pj-EE-BF), or water (Pj-EE-WF) in both in vitro (LPS-induced macrophage-like RAW264.7 cells) and in vivo (carrageenan-induced acute paw edema mouse models) experiments. The most active solvent fraction was selected for further analysis. Various in vitro and in vivo assessments, including nitric oxide (NO), cytokines, luciferase assays, real-time polymerase chain reactions, and immunoblotting analyses were performed to evaluate the underlying mechanisms. In addition, the phytochemical constituents were characterized by Liquid chromatography-tandem mass spectrometry. In in vitro studies, the highest inhibition of NO production was observed in Pj-EE-CF. Further examination revealed that Pj-EE-CF decreased the expression of inflammation-related cytokines in LPS-induced RAW264.7 cells and suppressed subsequent AP-1-luciferase activity by inhibition of phosphorylation events in the AP-1 signaling pathway. Pj-EE-CF treatment also demonstrated the strongest reduction in thickness and volume of carrageenan-induced paw edema, while Pj-EE-BF showed the lowest activity. Furthermore, Pj-EE-CF also reduced gene expression and cytokines production in tissue lysates of carrageenan-induced paw edema. These findings support and validate the evidence that Pj-EE, and especially Pj-EE-CF, could be a good natural source for an anti-inflammatory agent that targets the AP1 pathway.

## 1. Introduction

Inflammation is the protective response of the immune system against various pathogens and cellular danger signals, mediated primarily by immune cells such as macrophages. Acute inflammation is a process that involves the activation of complex enzymes, secretion of free radicals, and release of several inflammatory and pro-inflammatory mediators, which are commonly characterized by redness, swelling, pain, heat, and loss of tissue function [1,2,3]. The inflammatory response happens through the interaction of either pathogen-associated molecular patterns (PAMPs) or danger-associated molecular patterns with pattern recognition receptors [1,4]. For instance, engagement of PAMPs such as gram-negative bacteria-derived lipopolysaccharides (LPS) or carrageenan into toll-like receptor 4 will induce recruitment of adaptor proteins, MyD88 and TRIF, into the cytoplasm that eventually triggers inflammatory signaling cascades, including the activator protein-1 (AP-1) pathway [2,5,6]. The activation of the inflammatory signaling pathway is mediated by the initiation of signal transduction cascades activating a variety of intracellular signaling molecules. Three major mitogen-activated protein kinases (MAPKs), c-Jun N-terminal kinase (JNK), p38, and extracellular signal-regulated kinase (ERK), mediate nuclear translocation and activate transcription factors in the AP-1 pathway [3,7]. Activation of this transcription factor induces the expression of numerous inflammatory genes, such as inducible nitric oxide synthase (iNOS), cyclooxygenase-2 (COX-2), interleukin (IL)-6, IL-1β, and tumor necrosis factor-alpha (TNF-α), which will also stimulate matrix metalloproteinase (MMP) expression, subsequently releasing the production of the inflammatory mediator, nitric oxide (NO), and various cytokines [4,8,9,10,11]. Even though inflammation is a protective immune response, uncontrolled acute inflammation may become chronic, contributing to a variety of chronic inflammatory diseases. Several studies have provided evidence that inflammation is involved in the pathogenesis of many diseases including autoimmune diseases, cancer, and other life-threatening disorders [2,3,5,12]. As a result, a variety of efforts, including developing anti-inflammatory agents to regulate inflammatory responses and authenticating the agents to specifically target the molecular signaling, could potentially ameliorate several inflammatory diseases.

Some green algae (phylum *Chlorophyta*) are considered a representative natural source for pharmaceutical, nutraceutical, and cosmeceutical products [13,14]. Green algae, especially from the genus *Prasiola*, have a diverse distribution. At least 14 of 36 species are freshwater organisms, and other species grow in terrestrial habitats. *Prasiola japonica* belongs to the family *Prasiolaceae* and is found growing in freshwater ecosystems [15]. *Prasiola japonica* has been reported in east Asia and especially in Japan and in the Republic of Korea [16,17]. This alga possesses an assortment of components, including mannitol, loliolide, glucitol, alverine, diisopropylamine, and methyl pyrazine [18]. In recent years, the freshwater green algae *P. japonica* has been shown to possess medicinal benefits including antioxidant, antiapoptotic, anti-melanogenic, and anti-inflammatory effects. However, most of those studies were limited to identifying such effects of the crude ethanolic extract of *P. japonica* in the NF-κB pathway and mainly focused on the skin cell lines [13,18,19,20]. To the best of our knowledge, the present study is the first to describe the potential anti-inflammatory activities of *P. japonica* ethanol extract (Pj-EE) and four of its solvent fractions—hexane (Pj-EE-HF), chloroform (Pj-EE-CF), butanol (Pj-EE-BF), and water (Pj-EE-WF)—in in vitro (LPS-induced macrophage-like RAW264.7 cells) studies. The most active fraction, Pj-EE-CF, is selected to determine additional activities. In in vivo studies in carrageenan-induced acute paw edema mouse models, the molecular targets and phytochemical constituents of Pj-EE-CF are also evaluated.

## 2. Results

### 2.1. Effect of Pj-EE and Its Various Solvent Fractions on the NO Production of LPS-Induced Macrophages

**Figure 1:**
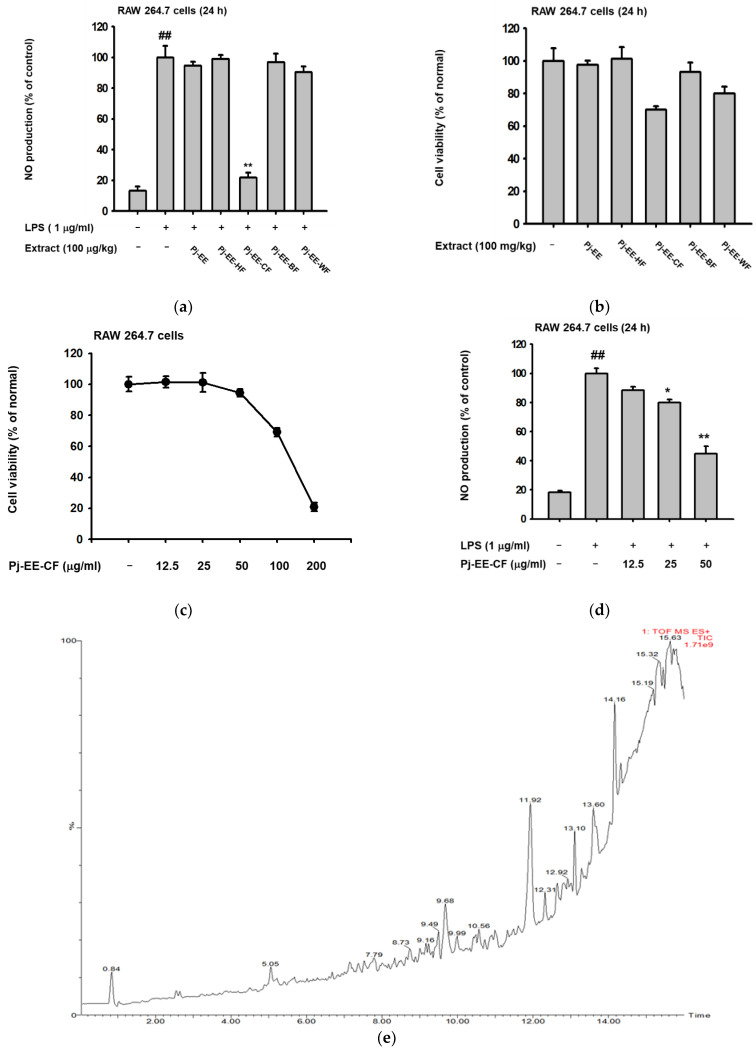
Effect of Pj-EE and its solvent fractions on the production of NO, cell viability profile, and phytochemical constituents of Pj-EE-CF. (**a**,**d**) Supernatant NO levels on LPS (1 μg/mL)-induced RAW264.7 cells pretreated with 100 μg/mL of Pj-EE, Pj-EE-HF, Pj-EE-CF, Pj-EE-BF, or Pj-EE-WF (**a**) and with the indicated concentrations of PJ-EE-CF (**d**) were analyzed using the Griess assay. (**b**,**c**) Cell viability of RAW264.7 cells upon treatment with Pj-EE, Pj-EE-HF, Pj-EE-CF, Pj-EE-BF, or Pj-EE-WF (**b**) and PJ-EE-CF (**c**) at the same concentration on NO assay were analyzed using an MTT assay. (**e**) The phytochemical screening performed on Pj-EE-CF using LC/MS-MS chromatogram. Results (**a**–**d**) are expressed as mean ± standard deviation. ^##^ *p* < 0.01 compared to normal group (no treatment), and * *p* < 0.05, ** *p* < 0.01 compared to control group (LPS alone) by one-way ANOVA.

To initially determine whether Pj-EE and its four solvent fractions have anti-inflammatory potential, we tested the production level of an inflammatory mediator such as NO on LPS-induced macrophages with the same concentrations of Pj-EE and its fractions (100 μg/mL). As demonstrated in Figure 1a, LPS significantly increased (*p* = 0.0021) the secretion of NO in macrophage-like RAW264.7 cells. Pj-EE-CF (100 μg/mL) had the strongest effect (*p* = 0.0071) on the secretion of NO and up to 80% inhibition on LPS-induced RAW264.7 cells. In contrast, treatment with Pj-EE, Pj-EE-HF, Pj-EE-BF, and Pj-EE-WF did not exert significant inhibition. Furthermore, using a conventional 3-(4,5-dimethylthiazol,2-yl)-2,5-diphenyltetrazolium bromide (MTT) assay, we checked the cell viability on macrophage-like RAW264.7 cells upon treatment with Pj-EE and the four solvent fractions. However, treatment with Pj-EE-CF at a concentration that significantly reduced NO production indicated an interference of cell viability by up to 25% (Figure 1b). Consequently, we sought to identify the potential effect of Pj-EE-CF at a lower concentration than 100 μg/mL that did not exert cytotoxicity. As shown in Figure 1c, treatment with Pj-EE-CF did not suppress cell viability at concentrations up to 50 μg/mL. Conversely, 100 and 200 μg/mL of Pj-EE-CF treatment significantly decreased cell viability by up to 80%. Furthermore, to check whether a concentration lower than 100 μg/mL of Pj-EE-CF also has anti-inflammatory activity, we re-evaluated the secretion of NO on LPS-induced in RAW264.7 cells. The result showed that pretreatment with Pj-EE-CF concentration-dependently and significantly suppressed secretion of NO production at concentration of 25 and 50 μg/mL (*p* = 0.0134 and *p* = 0.0044, respectively) (Figure 1d). These results suggested that treatment with Pj-EE-CF at concentrations up to 50 μg/mL affects NO production and has anti-inflammatory potential that is not due to cell death. Therefore, Pj-EE-CF was chosen to complete further experiments. Ultimately, we performed LC/MS-MS analyses to determine the phytochemical constituents of Pj-EE-CF, and we especially focused on parameter assays for flavonoids. Around 23 components from various flavonoid classes were observed, including the presence of maltol, at a retention time (RT) of 0.84, bavachinin at an RT of 6.11, flavonol at an RT of 6.42, 3’-deoxysappanone B at an RT of 8.20, kushenol N and X, at RTs of 10.61 and 11.62, respectively, nobiletin at an RT of 12.64, and phellochinin A at an RT of 14.53, as shown in Figure 1e and Supplementary Table S1.

### 2.2. Effects of Pj-EE-CF on the Expression of Pro-Inflammatory Genes on LPS-Induced Macrophages

**Figure 2:**
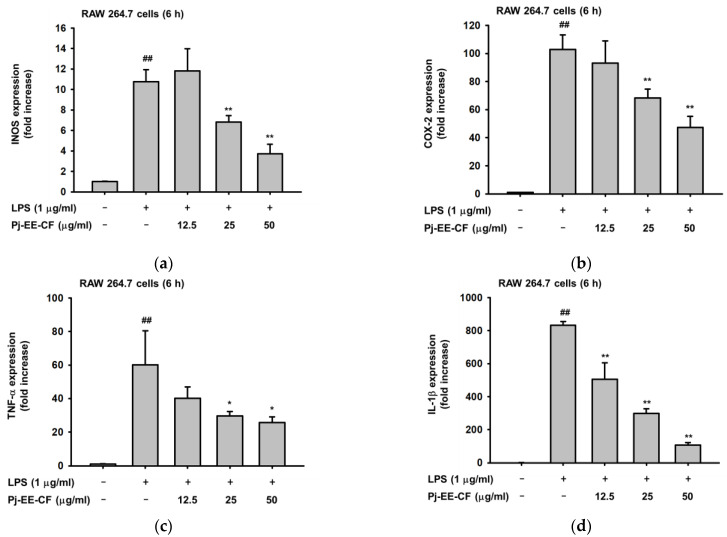

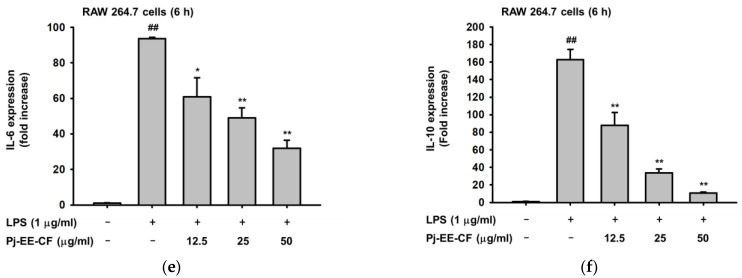
Effect of Pj-EE-CF on the expression of pro-inflammatory genes. The mRNA levels of iNOS (**a**), COX-2 (**b**), TNF-α (**c**), IL-1β (**d**), IL-6 (**e**), and IL-10 (**f**) were evaluated by qPCR on LPS-stimulated RAW264.7 cells. Results are expressed as mean ± standard deviation. ^##^ *p* < 0.01 compared to normal group, and * *p* < 0.05 and ** *p* < 0.01 compared to control group (LPS alone). The *p* value was calculated using Bio-Rad CFX software.

We initially determined that Pj-EE-CF treatment on LPS-stimulated RAW264.7 cells can reduce inflammatory mediators such as NO. To further our study, we examined whether Pj-EE-CF also can regulate the expression of pro-inflammatory mediators at the transcriptional level. Using quantitative real-time polymerase chain reaction (qPCR), we assessed the mRNA expression of iNOS, COX-2, and other pro-inflammatory cytokines, such as TNF-α, IL-1β, IL-6, and IL-10. As shown in Figure 2, LPS alone upregulated the level of inflammatory genes (*p* < 0.001), and treatment with Pj-EE-CF downregulated the expression of pro-inflammatory genes. In line with the result of the NO assay, LPS-stimulated RAW264.7 cells pretreated with Pj-EE-CF at concentrations of 25 and 50 μg/mL significantly inhibited in a concentration-dependent manner the mRNA levels of iNOS (*p* = 0.0094 and *p* = 0.0068, respectively), the enzyme that is responsible for catalyzing the secretion of NO (Figure 2a). Moreover, other pro-inflammatory and cytokine genes, such as COX-2, TNF-α, IL-1β, IL-6, and IL-10 (Figure 2b–f, respectively), also were shown to be reduced in a concentration-dependent manner by pretreatment with Pj-EE-CF, for concentrations of up to 50 μg/mL (*p* = 0.0026, *p* = 0.0211, *p* = 0.0005, *p* = 0.0022, and *p* = 0.0009, respectively). 

### 2.3. Effects of Pj-EE-CF on the Transcriptional Activation of AP-1 Signaling and the Upstream Signaling Molecules of AP-1 Activation

**Figure 3:**
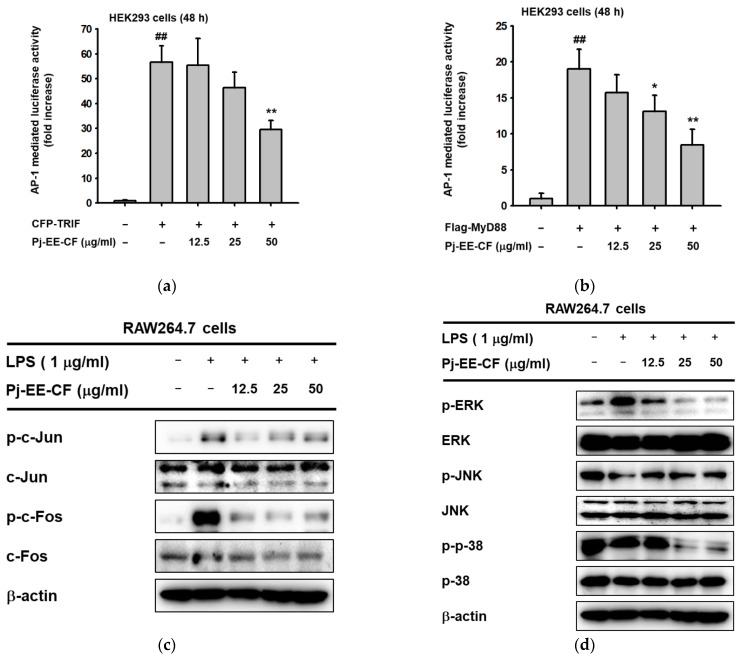
Effect of Pj-EE-CF on the transcriptional activation of AP-1 signaling and the upstream signaling molecules of AP-1 activation. (**a**,**b**) HEK293 cells were co-transfected with AP-1-Luc and β-gal (0.8 μg), as well as TRIF and MyD88 for 48 h in the presence or absence of Pj-EE-CF (12.5, 25, and 50 μg/mL), which were then examined using a luminometer. Results are expressed as mean ± standard deviation (*n* = 4). ^##^ *p* < 0.01 compared to normal group, * *p* < 0.05 and ** *p* < 0.01 compared to control group (LPS alone) by one-way ANOVA. (**c**) The phospho- and total forms of AP-1 subunits, c-Jun and c-Fos, from whole-cell lysates from LPS-treated RAW264.7 cells in the presence or absence of Pj-EE-CF (12.5, 25, and 50 μg/mL) were determined by immunoblot analysis. (**d**) RAW264.7 cells were pretreated with Pj-EE-CF (12.5, 25, and 50 μg/mL) for 30 min, followed by the presence or absence of LPS. The phosphorylated and total protein levels of ERK, JNK, and p38 were assessed by immunoblot analysis. β-actin was utilized as a control protein.

Next, due to the significant inhibitory effect of pro-inflammatory gene regulation, which was analyzed by qPCR, we sought to explore the transcriptional level using another assessment, the luciferase assay, using HEK293 cells co-transfected with either TRIF- or MyD88-mediated AP-1 activity. The results indicated that LPS alone significantly enhanced TRIF- or MyD88-mediated AP-1 activity (*p* = 0.0078 and *p* = 0.0035, respectively). However, treatment with up to 50 μg/mL of Pj-EE-CF concentration-dependently suppressed AP-1 luciferase activity induced by either TRIF or MyD88 (Figure 3a,b). In addition, we also examined the protein level of AP-1 subunit activation, specifically the phosphorylation of c-Jun and c-Fos, from whole lysates in LPS-induced RAW264.7 cells at different concentrations (12.5, 25, and 50 μg/mL). This finding showed that Pj-EE-CF decreased the level of p-c-Jun and p-c-Fos at all indicated concentrations (Figure 2c), which indicated that Pj-EE-CF could regulate the activity of AP-1 by inhibiting the dimerization of AP-1 via the reduction of activation of c-Jun and c-Fos. Afterward, to determine the target molecules of Pj-EE in the AP-1 pathway, we identified the effect of Pj-EE-CF at different concentrations in the upstream signaling molecules of AP-1 activation by an immunoblot analysis. MAPKs activation was generated by LPS-induced RAW264.7 cells. Among the MAPKs, the phosphorylation levels of ERK and of p38 were clearly decreased in the presence of Pj-EE-CF treatments at 25 and 50 μg/mL. However, the phosphorylation levels of JNK were not affected, despite the Pj-EE-CF treatment (Figure 2d). Taken together, these results suggested that Pj-EE-CF exerts anti-inflammatory responses through suppression of ERK and p38 in the AP-1 pathway. 

### 2.4. Effects of Pj-EE and Its Solvent Fractions on a Carrageenan-Induced Acute Paw Edema Model 

**Figure 4:**
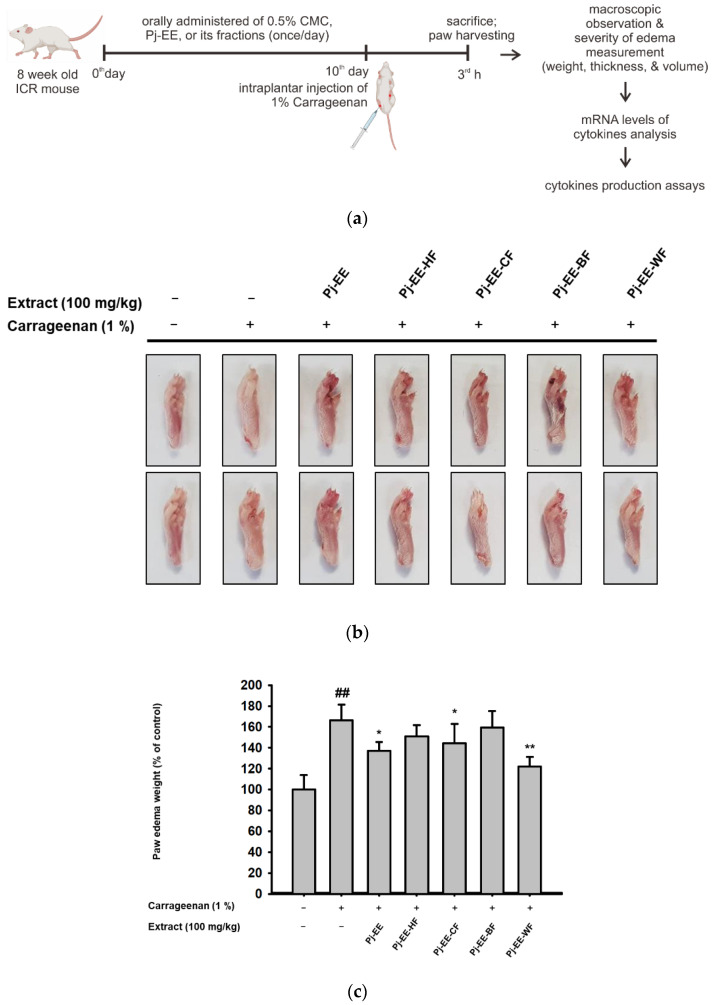

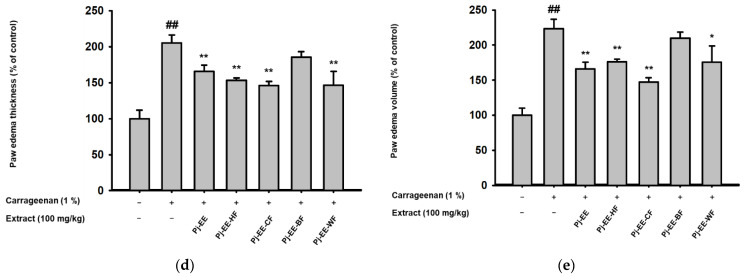
Effects of Pj-EE and four different solvent fractions on the carrageenan-induced acute paw edema mouse model. (**a**) Schematic of carrageenan-induced acute paw edema experiment. The ICR mice were orally treated with 100 μL of different solutions based on different groups for 10 days: a normal group (0.5% carboxymethylcellulose (CMC)) or carrageenan group as a control group (0.5% CMC), treatment groups (100 mg/kg Pj-EE, Pj-EE-HF, Pj-EE-CF, Pj-EE-BF, or Pj-EE-WF). Acute paw edema was triggered by an intraplantar injection of 1% carrageenan (100 μL/mouse) and mice were sacrificed after 3 h. (**b**) A representative photograph of paw inflammatory symptoms. (**c**–**e**) The severity of paw edema was evaluated by measuring the weight (**c**), thickness (**d**), and volume (**e**) of each paw of mouse. Results are expressed as mean ± standard deviation (*n* = 12). ^##^ *p* < 0.01 compared to normal group, and * *p* < 0.05 and ** *p* < 0.01 compared to control group (carrageenan alone). The *p*-value was analyzed using one-way ANOVA.

To determine the anti-inflammatory efficacy of Pj-EE and its solvent fractions in vivo, carrageenan-induced acute paw edema mouse models were used. Inflammation in both hind paws of each mouse was triggered by intraplantar injection of 1% newly prepared solution of carrageenan in PBS after pretreatment with 100 mg/kg Pj-EE and the four different solvent fractions once a day for 10 days, as illustrated in Figure 4a. As expected, carrageenan-induced paw edema showed prominent inflammatory symptoms, such as redness and swelling of the paws (Figure 4a). In contrast, 100 mg/kg of Pj-EE and its solvent fractions decreased the symptoms compared to the control group (carrageenan alone). In addition, we quantitatively measured the weight, thickness, and volume (Figure 4b–d) of each mouse paw to compare the severity of edema in each group. The water fraction following treatment with Pj-EE and Pj-EE-CF showed a significant reduction in paw weight compared to the control group (*p* = 0.0098, *p* = 0.0477, and *p* = 0.0354, respectively). However, Pj-EE-CF had the lowest measurement both of thickness and volume compared to 100 mg/kg of Pj-EE or the other solvent fraction groups, which suggested that it had more potent inhibitory effects, followed by Pj-EE, Pj-EE-HF, and Pj-EE-WF. Additionally, Pj-EE-BF did not show inhibitory symptoms on the carrageenan-induced paw edema mouse model.

### 2.5. Effects of Pj-EE and Its Solvent Fractions on Pro-Inflammatory Genes and Cytokines Production in Carrageenan-Induced Acute Paw Edema Models

**Figure 5:**
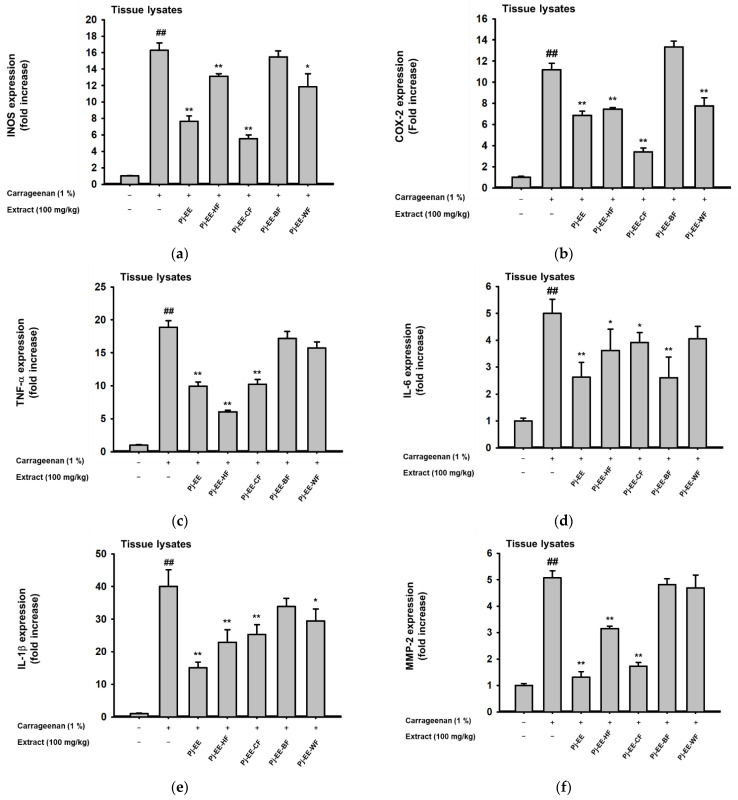

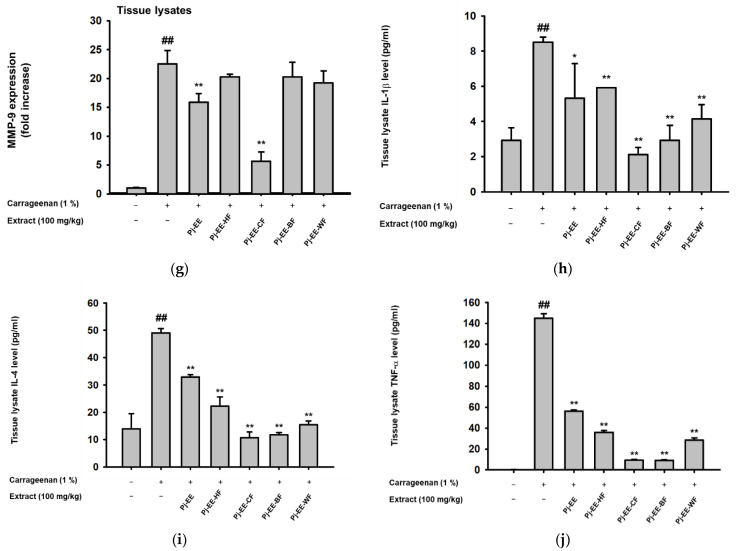
Effects of Pj-EE and four solvent fractions (HF, CF, BF, and WF) on the pro-inflammatory genes and cytokines production in carrageenan-induced acute paw edema models. (**a**–**g**) The expression of inflammatory genes, including iNOS (**a**), COX-2 (**b**), TNF-α (**c**), IL-6 (**d**), IL-1β (**e**), MMP-2 (**f**), and MMP-9 (**g**) from paw lysates was measured by quantitative real-time PCR. (**h**–**j**) Supernatant IL-1β (**h**), IL-6 (**i**), and TNF-α (**i**) levels of tissue lysates on carrageenan-triggered paw edema were examined by EIA. All of the data are expressed as mean ± standard deviation (*n* = 6). ^##^ *p* < 0.01 compared to the normal group, and * *p* < 0.05 and ** *p* < 0.01 compared to the control group (inducer alone) by one-way ANOVA.

To evaluate the effects of Pj-EE and its solvent fractions at the molecular level, the level of inflammatory genes in the tissue lysate of paw samples was examined by qPCR (Figure 5a–g). In agreement with in vitro experiments, Pj-EE-CF possessed the highest inhibition of inflammatory genes, such as iNOS (Figure 5a), COX-2 (Figure 5b), and MMP-9 (Figure 5g), whereas Pj-EE-BF had the lowest, or barely any, activities. Additionally, for other pro-inflammatory genes such as TNF (Figure 5c), Pj-EE-HF showed more significantly reduced expression of TNF-α, followed by Pj-EE and Pj-EE-CF. In addition, in terms of the expression of IL-6 (Figure 5d), IL-1β (Figure 5e and MMP-2 (Figure 5f), the crude Pj-EE showed more prominent inhibition of the inflammatory genes, despite Pj-EE-CF still showing significant inhibition activities of these pro-inflammatory genes. To confirm that Pj-EE and its solvent fractions effectively inhibited the secretion of various cytokines, we further examined the production of IL1-β, IL-4, and TNF-α using enzyme immunoassay (EIA). Carrageenan-induced paw edema significantly generated the production of these cytokines, but treatment with 100 mg/kg PJ-EE and its four solvent fractions showed a reduction of these enzymes, with Pj-EE-CF displaying the highest attenuation of IL1-β (Figure 5h), IL-4 (Figure 5i), and TNF-α (Figure 5j) secretions. These data indicated that Pj-EE alleviated the inflammatory symptoms on carrageenan-induced paw edema, and, notably, Pj-EE-CF conferred more potent inhibition of almost all the assessments. 

## 3. Discussion 

Medicinal plants with natural sources still offer promising options and are recognized as therapeutic agents. In addition, understanding the mechanisms of therapeutic action could lead to the discovery of new drugs from natural sources with reduced or no side effects [21,22,23]. The edible freshwater green algae, *P. japonica*, has been described to possess pharmacological benefits. Last year our research group reported that the ethanol extract of *P. japonica* exerts antiapoptotic, anti-melanogenic, and antioxidant effects on skin cell lines [13,19]. We also recently showed in vivo anti-inflammatory effects via the NF-κB signaling pathway [20]. However, additional studies with respect to which solvent fractions have potential activity remained unclear. Therefore, in this study, using Pj-EE extract and four different solvent fractions, we aimed to investigate their anti-inflammatory potential against LPS-induced macrophage-like RAW264.7 cells and in carrageenan-induced paw edema mouse model, and then focused on which solvent fraction possessed more potent activities. We also determined the molecular mechanisms concerning the AP-1 pathway and the phytochemical constituents.

In early characterizations of NO, it was described as playing an important role in biological activities such as neurotransmission and immune defenses. However, research showed that while the production of NO is uncontrolled in the cells, it acts as one of the pro-inflammatory mediators and a key molecule during inflammatory responses [8]. In this study, NO production was stimulated by LPS, a major component of the outer membrane of gram-negative bacteria. Aggregation of the TLR4-adaptor proteins complex after binding LPS led to activation of multiple signaling molecules, and the subsequent production of pro-inflammatory cytokines and mediators [3,24]. Our first finding revealed that Pj-EE-CF showed the highest inhibition of the secretion of NO (Figure 1). Based on this finding, we focused our investigations on its molecular mechanisms and also described its phytochemical constituents. Pj-EE-CF inhibited the expression of iNOS, an enzyme responsible for the mediation of NO production, in LPS-induced macrophages [8]. Other inflammatory cytokines and mediators that are broadly involved in inflammation via activation of its transcription, including COX-2, TNF-α, interleukins, and MMPs, were also evaluated. The mRNA levels of those related genes, analyzed by qPCR, were determined to be downregulated in a concentration-dependent manner by Pj-EE-CF in vitro and in vivo (Figure 2 and Figure 5), suggesting that Pj-EE-CF could regulate inflammatory responses at the transcriptional level. Importantly, certain concentrations of Pj-EE-CF produced these effects without affecting cell viability, which suggested that the inhibitory effects were not due to non-specific toxicity. 

In addition, to precisely identify the molecular target and mechanism of Pj-EE-CF, other experimental approaches, including luciferase assays and immunoblot analyses, were performed. The results of the luciferase reporter genes indicated that Pj-EE-CF regulates the mRNA level of cytokines by moderating the transcriptional activity of AP-1. Strengthening these findings, immunoblot analyses of the level of phosphorylation of AP-1 subunits, c-jun and c-Fos, revealed that Pj-EE-CF modulates the transcriptional activation of AP-1. Previous studies reported the essential role and mechanism of MAPKs in activating AP-1 pathways [7,23,25]. Consequently, we checked signaling molecules upstream of this AP-1 activation, and Pj-EE-CF specifically inhibited ERK and p38 activation. These results indicated how effective Pj-EE-CF can be in attenuating anti-inflammatory responses, resulting in a reduction of ERK and p38 of the AP-1 pathway (Figure 3).

Several studies on the discovery and development of anti-inflammatory agents were based on the use of carrageenan-induced inflammation. Carrageenan-induced paw edema has been established as an experimental model of acute inflammatory diseases and has been widely used for studying novel analgesic and anti-inflammatory agents [6,20,26,27,28]. Carrageenan injection triggered an innate immune response characterized by edema, redness, and the continuing of neutrophil infiltration, and pro-inflammatory mediators and cytokines were involved in this process through TLR4-MyD88 or the TRIF complex [6]. Accordingly, to further evaluate and screen the anti-inflammatory efficacy of Pj-EE and its solvent fractions in vivo, we employed a carrageenan-induced paw edema mouse model. In agreement with other reported studies, intraplantar injection of 1% carrageenan into the hind paw led to inflammatory symptoms in the paws and generated the expression and production of pro-inflammatory cytokines. In addition, consistent with our in vitro results, Pj-EE treatment, and especially PJ-EE-CF treatment, decreased inflammatory symptoms and exhibited protective effects for carrageenan-induced paw edema (Figure 4). Furthermore, because carrageenan has been reported to induce the production of pro-inflammatory cytokines, such as TNF-α, IL-1β, and IL-4, we also determined the production of these cytokines by enzyme immunoassay. The results indicated that Pj-EE and its four solvent fractions decreased cytokines production, with Pj-EE-CF showing the most significant inhibition (Figure 5). Taken together, the results supported that orally administered Pj-EE and its solvent fractions, especially Pj-EE-CF, were able to alleviate inflammatory responses and could be clinically beneficial for treating inflammatory symptoms. 

Phytochemical screening, especially pertaining to the flavonoids, for the possible presence of Pj-EE-CF was characterized using liquid chromatography-tandem mass spectrometry (LC-MS/MS). Numerous flavonoid molecules have been described to obtain anti-inflammatory activity through various mechanisms, including inhibition of the expression of inflammation-related enzymes via suppression of transcription factors activation, including AP-1 [29,30,31]. Among the various flavonoid types that were present, maltol, bavachinin, nobiletin, flavonol, and 3′-deoxysappanone B have been widely reported to possess bioactivity, such as anticancer, antimicrobial, and anti-inflammatory properties. The major flavonols, such as quercetin, kaempferol, isorhamnetin, and galangin, were found to exhibit anti-inflammatory activity [29,31]. Maltol exerts a significant liver protection effect, and possesses anti-inflammatory and antiapoptotic actions [32]. Some studies demonstrated that bavachinin, nobiletin, and 3′-deoxysappanone B regulate the production of several cytokines as well as inflammatory mediators in activated macrophages and various other cell types [32,33,34,35,36,37,38]. Consequently, we evaluated whether these components of Pj-EE-CF could be responsible for its anti-inflammatory effect.

## 4. Materials and Methods

### 4.1. Materials

Macrophage-like RAW264.7 cells (ATCC number TIB-71) and HEK293 cells (ATCC number CRL-1573) were obtained from the American Type Culture Collection (ATCC) (Rockville, MD, USA). TRIzol reagent was purchased from MRCgene (Cincinnati, OH, USA). Carrageenan, dimethyl sulfoxide (DMSO), carboxymethylcellulose (CMC), lipopolysaccharide (LPS, Escherichia coli 0111:B4), polyethylenimine (PEI), sodium dodecyl sulfate, and 3-(4,5-dimethylthiazol,2-yl)-2,5-diphenyltetrazolium bromide (MTT) were purchased from Sigma-Aldrich Co. (St. Louis, MO, USA). Fetal bovine serum was acquired from Biotechnics Research, Inc. (Irvine, CA, USA). RPMI 1640, DMEM, trypsin, PBS, and penicillin-streptomycin were obtained from HyClone (Logan, UT, USA). Primers used for qPCR were obtained from Macrogen Inc. (Seoul, Korea). Phospho-specific or total-protein antibodies against c-Fos, c-Jun, JNK, ERK, p38, and β-actin were purchased from Cell Signaling Technology (Beverly, MA, USA) and Santa Cruz Biotechnology (Santa Cruz, CA, USA). A CATX124 balance was purchased from CAS Co. (Yangju, Korea), a Mitutoyo thickness gauge 547 was received from Mitutoyo (Kanagawa, Japan), and a plethysmometer 37,140 was obtained from UgoBasile (Comerio, VA, Italy). 

### 4.2. Pj-EE and Preparation of Its Solvent Fractions 

**Figure 6:**
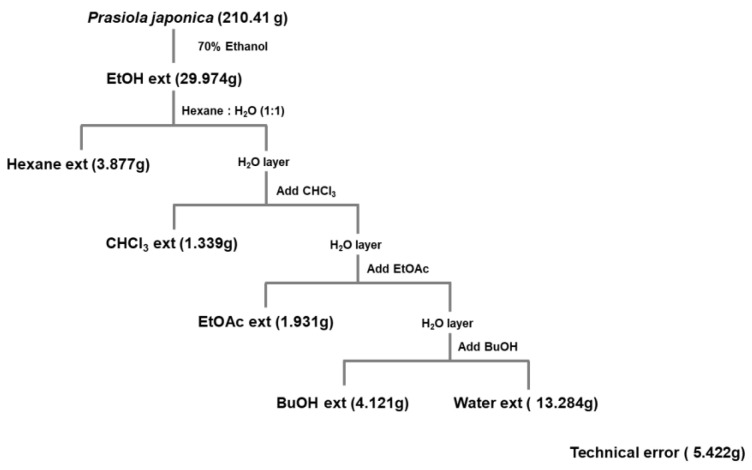
Schematic: preparation of ethanolic extract and four solvent fractions of *Prasiola japonica*.

*P. japonica* was acquired from the Prasiola Japonica Research Center (Samcheok City, Gangwon-do, Republic of Korea) and was extracted as in previous studies [13,19,20]. Briefly, pieces of cut samples were extracted with 70% ethanol for 24 h at a ratio of 1:20 (*w*/*v*). Subsequently, the extract was filtered using 120 nm filter paper (No. 2, Advantec, Toyo Co., Tokyo, Japan), concentrated using a vacuum concentrator (Eyela New Rotary Vacuum Evaporator, Rikakikai Co., Tokyo, Japan), and then dried using a vacuum freeze dryer (Eyela FD1, Rikakikai Co.). The yield of the dried samples was measured. The final weight of the extract was 29.974 g (original sample: 210.41 g) with a yield of 14.24%. In addition, the ethanolic extract of P. japonica (crude extract) was then fractionated by various polarity solvents including n-hexane (Pj-EE-HF), chloroform (Pj-EE-CF), n-butanol (Pj-EE-BF), and water (Pj-EE-WF) as illustrated in Figure 6. The dried samples were kept in a −20 °C freezer for future use. For the in vitro studies, stock solutions of Pj-EE and of its solvent fraction were prepared by dissolving them with DMSO at a concentration of 100 mg/mL. When each experiment was performed, the stock solution was diluted to the desired final concentration of 12.5, 25, 50, 100, or 200 μg/mL using the suitable culture medium. For the paw edema mouse model experiments, the stock solution was made in 0.5% CMC at doses of 100 mg/kg.

### 4.3. Cell Culture and Treatment 

The murine macrophage cell line (RAW264.7) and the human embryonic kidney cell line (HEK293) were cultured as previously reported [39]. For a preliminary study, LPS-induced RAW264.7 cells were pretreated at a concentration of 100 μg/mL of Pj-EE and its solvent fractions, whereas for the next experiments, LPS-activated RAW264.7 cells were pretreated with Pj-EE-CF at concentration 12.5, 25, and 50 μg/mL. The control (LPS alone) and normal groups were pretreated with diluted DMSO in culture medium for 30 min. The final concentration of DMSO in the cellular experimental conditions was <0.5%.

### 4.4. Determination of NO and Cytokines Production

RAW264.7 cells (1 × 10^6^ cells/mL) were seeded in a 96-well plate and then pretreated with the specified concentrations of Pj-EE and its solvent fractions for 30 min. LPS (1 μg/mL) was then treated for the next 24 h. The suppression effect of Pj-EE and its solvent fractions on the secretion of NO was analyzed using Griess reagents, as previously described [40]. The suppression effects of Pj-EE and its solvent fractions on the secretion of IL-1β, IL-4, and TNF-α were detected using an EIA kit according to the manufacturer’s instructions (R&D Systems, catalog no. MLB00C, M4000B, and MTA00B, respectively). Briefly, after diluting the supernatant in the indicated assay diluent 1:1, 100 μL was added to each well in a 96-well plate coated with anti-mouse IgG. After washing 5 times with wash buffer (400 μL), 100 μL of mouse IL-1β, IL-4, or TNF-α conjugate was added to each well and incubated for 2 h. After the reaction and washing, 100 µL of substrate solution was added to each well and incubated for an additional 30 min. The incubation was stopped with 100 μL of the substrate solution and was measured at 450 nm.

### 4.5. Cell Viability Assay

The cytotoxic effects of Pj-EE and its solvent fractions were determined by a conventional MTT assay as previously reported [41]. We seeded RAW264.7 cells into 96-well plates and preincubated the overnight (16 h). Afterwards, we treated them with the indicated concentrations of Pj-EE and its solvent fractions or DMSO (for the normal group) for an additional 24 h.

### 4.6. Liquid Chromatography-Tandem Mass Spectrometry (LC-MS/MS)

The phytochemical screening of Pj-EE-CF was characterized by LC-MS/MS. LC-MS/MS analyses were performed as previously described [23].

### 4.7. mRNA Analysis by Quantitative Real-Time Polymerase Chain Reaction (qPCR)

Total RNA was obtained from macrophage-like RAW264.7 cells (1 × 10^6^ cells/mL) that were pretreated with Pj-EE-CF (0, 12.5, 25, and 50 μg/mL) for 30 min followed by induction with LPS (1 μg/mL) for 6 h. Paw tissues of mice were ground, and then stored at −70 °C until use. Total RNA was prepared using TRIzol per the manufacturer’s instructions, and then 1 μg of total RNA was immediately made for cDNA synthesis using a cDNA synthesis kit (Thermo Fisher Scientific, Waltham, MA, USA) per the manufacturer’s instructions. The qPCR was performed as previously reported [21]. The primer sequences used in this study are listed in Table 1.

### 4.8. Luciferase Reporter Gene Assay

HEK293 cells (2 × 10^5^ cells/mL) were seeded in 24-well plates and preincubated overnight before transfection of plasmids encoding AP-1-Luc under cotransfection conditions with MyD88 and TRIF, by PEI methods as previously reported [23]. After 24 h transfection, the cells were treated with 12.5, 25 or 50 μg/mL of Pj-EE-CF for an additional 24 h.

### 4.9. Cell Lysate Extraction and Immunoblotting Analysis

RAW264.7 cells (1 × 10^6^ cells/mL) were seeded in a 6-well plate. After pretreatment with different concentrations of PJ-EE-CF for 30 min, LPS was treated and then incubated for the next 24 h. After harvesting and washing with PBS, RAW264.7 cells were lysed in lysis buffer as previously described [42]. Approximately 20 μg of protein were subjected to western blot analysis as previously described [23].

### 4.10. Animals

ICR mice (8 weeks old, 20–22 g, male) were purchased from Daehan Biolink (Chungcheonbuk, Korea). The mice (*n* = 6 per group) had water and a pellet diet (Samyang, Daejeon, Korea) ad libitum in separate cages under a 12 h light–dark cycle. The in vivo experiments were conducted in agreement with the guidelines of the Institutional Animal Care and Use Committee, Sungkyunkwan University (Suwon, Korea; approval ID: SKKUIACUC2020-06-39-1).

### 4.11. Carrageenan-Induced Acute Paw Edema Mouse Model

Using ICR mice (6 mice/group), paw edema was stimulated by a 100 μL subplantar injection of 1% newly made solution of carrageenan in PBS into both hind paws, as in the previously published report [27]. ICR mice were orally administrated with 100 μL of specific solutions for the different groups: normal group (0.5% CMC), carrageenan alone as control group (0.5% CMC), and treatment groups (100 mg/kg Pj-EE and its solvent fractions) once a day for 10 days. After that, carrageenan-induced paw edema was apparently observed in each group except the normal group (subplantar injection with PBS). After 3 h, the mice were sacrificed by CO_2_. Subsequently, redness and swelling was observed in both paws. The weight, thickness, and volume of the edema were measured as described previously [20].

### 4.12. Statistical Analysis

All data in this study represent the mean ± standard deviation of four samples (in vitro experiments). Similar experimental data were obtained from additional independent experiments performed under the same conditions with six mice per group (in vivo experiments). Statistical analyses was performed using the computer program SPSS (version 26, SPSS Inc., Chicago, IL, USA). A comparison of statistical differences of all measured data was subjected to one-way ANOVA followed by the Holm–Sidak test or the Kruskal–Wallis/Mann–Whitney test. A *p*-value of <0.05 was considered to be statistically significant. 

## 5. Conclusions

**Figure 7:**
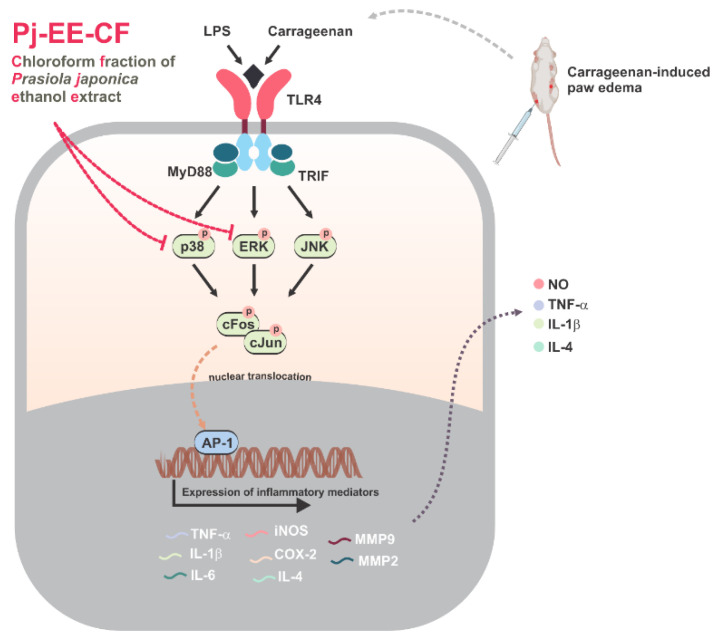
Schematic: anti-inflammatory mechanisms of Pj-EE-CF in the suppression of AP-1 signaling pathway.

The present study, using in vitro and in vivo experiments, clearly indicated and supported the evidence of the anti-inflammatory effects of Pj-EE. Pj-EE-CF showed a particularly potent inhibition of inflammatory responses by targeting ERK and p38, thereby suppressing the activity of AP-1 that could result in attenuation of various inflammatory mediators and cytokines, as summarized in Figure 7. The anti-inflammatory activity of Pj-EE-CF is provided in the context of bioactive molecules that have demonstrated this function in previous studies. In the future, Pj-EE could be a good natural source of an anti-inflammatory agent, especially Pj-EE-CF. Further investigations are necessary to evaluate and isolate the bioactive molecules present in Pj-EE-CF.

## Figures and Tables

**Table 1:** Primer sequences used for quantitative real-time PCR.

PCR Type	Genes Name	Sequence (5′-3′)	
q PCR	GAPDH	Forward	GAAGGTCGGTGTGAACGGAT
		Reverse	AGTGATGGCATGGACTGTGG
	iNOS	Forward	CAAGAGAACGGAGAACGGAGA
		Reverse	GATGGACCCCAAGCAAGACT
	COX-2	Forward	TGAGTACCGCAAACGCTTCT
		Reverse	TGGGAGGCACTTGCATTGAT
	TNF-α	Forward	TTGACCTCAGCGCTGAGTTG
		Reverse	CCTGTAGCCCACGTCGTAGC
	IL-1β	Forward	CAGGATGAGGACATGAGCACC
		Reverse	CTCTGCAGACTCAAACTCCAC
	IL-6	Forward	GCCTTCTTGGGACTGATGCT
		Reverse	TGGAAATTGGGGTAGGAAGGAC
	IL-4	Forward	GGTCTCAACCCCCAGCTAGT
		Reverse	GCCCATGATCTCTCTCAAGT
	MMP-2	Forward	GTCCCTACCGAGTCTCTTCT
		Reverse	TTTTTAAGTTTCCGCTTCTG
	MMP-9	Forward	GCCACTTGTCGGCGATAAGG
		Reverse	CACTGTCCACCCCTCAGAGC

## Data Availability

The data used to support the findings of this study are available from the corresponding author upon request.

## Supplementary Materials

Supplementary Table S1: LC-MS/MS of Pj-EE-CF.

## Sample Availability

Samples of the compound, Pj-EE-CF, are available from the authors.

## Abbreviations

LPS	Lipopolysaccharide
MyD88	Myeloid differentiation factor 88
AP-1	Activator protein-1
MAPKs	Mitogen-activated protein kinases
JNK	c-Jun N-terminal kinase
RPMI 1640	Roswell Park Memorial Institute 1640
DMEM	Dulbecco’s modified Eagle’s medium
COX-2	Cyclooxygenase-2
MMP	Matrix metalloproteinase
IL-1β	Interleukin-1β
IL-6	Interleukin 6
TNF-α	Tumor necrosis factor alpha
MKK	Mitogen-activated protein kinase
iNOS	Inducible nitric oxide synthase
