# Supplementary material for: Anti-Inflammatory Activities of the Ethanol Extract of Prasiola japonica, an Edible Freshwater Green Algae, and Its Various Solvent Fractions in LPS-Induced Macrophages and Carrageenan-Induced Paw Edema via the AP-1 Pathway
Source: Molecules. 2021 Dec 29;27(1):194. doi: 10.3390/molecules27010194 (PMC8746635; doi:10.3390/molecules27010194)
Supplement: Supplementary file 1 [file molecules-27-00194-s001.zip › molecules-1473655-supplementary.pdf]

PJ-EE-CF												
**No.**	**Component name**	**Formula**	**Observed RT (min)**	**Mass error (ppm)**	**Total Fragments Found**	**Isotope Match Mz RMS PPM**	**Isotope Match Intensity RMS Percent**	**Detector counts**	**Response**	**Adducts**	**Neutral mass (Da)**	**Observed neutral mass (Da)**
215	Maltol	C6H6O3	0.84	1.9	7	71.04	4893.66	32978	9963	Sn	126.03169	126.0322
42	4,7,2′-Trihydroxy-4′-methoxyisoflavanol	C16H16O5	1.91	-0.8	0	1.55	112.83	33278	28193	+Li	288.09977	288.0995
183	Kushenol N	C26H30O7	10.61	3.2	25	5.87	44.19	76520	53852	+H	454.19915	454.2006
140	Isosakuranetin	C16H14O5	10.86	3.9	31	5.86	86.40	19297	11947	+NH4	286.08412	286.0853
197	Kushenol X	C25H28O7	11.62	-3.7	10	5.27	26.28	200960	149996	+H	440.18350	440.1819
214	Mahuannin J	C30H20O11	11.87	-4.8	2	21.80	903.49	13166	13166	+H	556.10056	556.0979
129	Isoanhyoicaritin	C22H24O6	12.32	1.9	92	2.56	35.82	44567	29318	+H	384.15729	384.1580
227	Nobiletin	C21H22O8	12.64	-2.6	33	3.47	580.39	14081	14081	+H	402.13147	402.1304
127	Icariside-I	C27H30O11	14.32	0.9	88	2.73	951.72	12536	12536	+H	530.17881	530.1793
233	Phellochinin A	C26H30O11	14.53	2.4	62	3.48	11460.98	160009	160009	+Na	518.17881	518.1801
239	Polygalaxanthone Ⅲ	C25H28O15	2.87	-0.1	1	2.56	561.12	7949	7949	+Li	568.14282	568.1427
39	3-O-[β-D-Glucopyra-nosyl-(1→2)]-β-D-glucopyranosyl-kaempferol	C27H30O16	3.72	0.2	10	1.21	789.14	237764	161541	+Li, +Na	610.15338	610.1535
220	Nelumboroside A	C27H30O16	3.98	-0.7	10	1.37	199.38	51405	40152	+Li	610.15338	610.1530
30	2''-O-Acetylrutin	C29H32O17	4.91	-0.1	18	0.32	6.86	696911	431915	+Li, +Na	652.16395	652.1639
78	Bavachromene	C20H18O4	5.00	1.7	6	1.96	1020.47	323062	271920	+Li	322.12051	322.1211
206	Leucodelphinidin	C15H14O8	5.53	3.2	0	3.81	142.39	7021	7021	+H	322.06887	322.0699
48	6,7-Dihydroxy-2-(2-phenylethyl) chromone	C19H18O4	5.59	-4.7	4	5.18	23.24	35148	30108	+H	310.12051	310.1190
77	Bavachinin	C21H22O4	6.11	0.1	7	1.53	180.50	54961	44931	+Li	338.15181	338.1518
16	2′,6′-Dihydroxy-4,4′-dimethoxydihydrochalcone	C17H18O5	6.17	2.3	3	2.33	993.23	303090	256994	+Li	302.11542	302.1161
106	Flavonol	C15H10O3	6.42	0.8	1	300.66	122.80	39878	39878	+Li	238.06299	238.0632
11	(3R,4S)-3,4-Dihydroxy-3-(3′,4′-dimethoxybenzyl)-7-methoxy-chroman	C19H22O6	6.51	2.7	6	5.68	227.44	66169	53923	+Li	346.14164	346.1426
7	(3R,4S)-3,4-Dihydroxy-3-(3′,4′-dimethoxybenzyl)-7-methoxy-chroman	C19H22O6	7.05	-3.6	16	4.39	303.59	210037	8752	+Li	346.14164	346.1404
38	3'-Deoxysappanone B	C18H18O5	8.20	-1.4	9	1.52	25.27	45743	38102	+Na	314.11542	314.1149
